# Early start of growth hormone is associated with positive effects on auxology and metabolism in Prader-Willi-syndrome

**DOI:** 10.1186/s13023-020-01527-0

**Published:** 2020-10-12

**Authors:** Lucy Magill, Constanze Laemmer, Joachim Woelfle, Rolf Fimmers, Bettina Gohlke

**Affiliations:** 1 Department of Pediatric Endocrinology and Diabetology, Children’s Hospital, University of Bonn, Venusberg-Campus, Building 30, 53127 Bonn, Germany; 2 Pediatric Endocrinology and Diabetology, St. Bernward Hospital, Treibestraße 9, 31134 Hildesheim, Germany; 3 Children’s University Hospital Erlangen, Loschgestrasse 15, 91054 Erlangen, Germany; 4 University Hospital of Bonn, Institute for Medical Biometry, University of Bonn, Venusberg-Campus, 53127 Bonn, Germany

**Keywords:** Prader-Willi-syndrome, Growth hormone therapy, Carbohydrate and lipid metabolism, Insulin-like growth factor-I

## Abstract

**Background:**

Prader-Willi-Syndrome (PWS) is characterized by hypothalamic-pituitary dysfunction. Recent research suggests starting growth hormone-treatment (GHT) as soon as possible. The aim of this study is to analyze possible differences in auxological parameters, carbohydrate and lipid metabolism between two groups of children with PWS that started GHT either during or after their first year of life.

**Study design:**

Retrospective longitudinal study of 62 children (31 males) with genetically confirmed PWS. Upon diagnosis all children were offered GHT, some started immediately, others commenced later. Cohort A (*n* = 21; 11 males) started GHT at 0.3–0.99 yrs. (mean 0.72 yrs) and Cohort B (*n* = 41; 20 males) commenced GHT at 1.02–2.54 yrs. (mean 1.42 yrs) of age. Fasting morning blood samples and auxological parameters were obtained before the start of therapy and semi-annually thereafter. Differences between the two cohorts were estimated with a linear mixed-effect model.

**Results:**

Mean length/height-SDS_PWS_ differed significantly between the groups [1 yr: A: 0.37 (±0.83) vs B: 0.05 (±0.56); 5 yrs.: A: 0.81 (±0.67) vs B: 0.54 (±0.64); *p* = 0.012]. No significant differences were found in BMI, lean body mass or body fat.

Low-density cholesterol was significantly lower in A than in B [LDL: 1 yr: A: 79 (±20) mg/dl vs B: 90 (±19) mg/dl; 5 yrs.: A: 91(±18) mg/dl vs 104 (±26) mg/dl; *p* = 0.024].

We found significant differences in the glucose homeostasis between the groups [fasting insulin: *p* = 0.012; HOMA-IR: *p* = 0.006; HbA1c: *p* < 0.001; blood glucose: *p* = 0.022].

**Conclusions:**

An early start of GHT during the first year of life seems to have a favorable effect on height-SDS and metabolic parameters.

## Background

Prader-Willi-Syndrome (PWS), first described by Prader, Willi, and Labhart in 1956 [1], is a rare multisystem genetic disorder characterized by hypothalamic-pituitary dysfunction [2]. The population prevalence of this disorder has been estimated between 1/10,000–1/30,000 [3]. Characteristic features in children with PWS are severe neonatal hypotonia and feeding difficulties with failure to thrive, an early onset of hyperphagia with food seeking behavior and, accordingly, with a progressive development of severe obesity, an abnormal body composition and a short stature, a delayed overall development with cognitive deficiency and behavioral abnormalities [3–6]. Several endocrine problems such as hypogonadism, hypothyroidism, growth hormone (GH) and adrenal deficiency have been described [7–10]. PWS is caused by a lack of expression of paternal genes from chromosome 15q11.2-q13 [11]. There are four main mechanisms causing this absence of expression: paternal deletion (65–75%), maternal uniparental disomy (20–30%), and imprinting defects or balanced chromosome 15 translocations (1–3%) [11–13]. Since growth hormone treatment (GHT) was approved in Europe for PWS in 2001, the physical benefits of the treatment have been investigated in many randomized and controlled studies [14]. Findings from these studies show that GHT may improve bone mineral density, body composition, growth, head circumference and lipid profiles in patients with PWS [15–21]. Studies have also shown cognitive benefits and an improvement in long-term health-related quality of life [22, 23]. In the past, a start of GHT during early childhood, especially before the onset of obesity at around 2 years of age, was widely recognized as beneficial [14]. More recent findings demonstrate that additional benefits can be obtained when treatment is started from as early as 2 to 6 months of life age [19, 24, 25]. Thus, starting GHT earlier during the first year of life and/or immediately after the diagnosis of PWS has become increasingly popular [14]. It is now recommended to start treatment as soon as possible [7], yet studies testing these recommendations are still scarce. The aim of this study is to analyze whether there are differences in auxological parameters, carbohydrate and lipid metabolism between children with PWS who started GHT either during or after their first year of life.

## Methods and patients

### Methods

#### Anthropometry and body composition

Length and height were measured with a rigid stadiometer and the children’s weight was measured while unclothed to the nearest 0.1 kg on a mechanical scale. Body mass index (BMI) was determined using the formula kilograms/height^2^. Standard deviation scores (SDS) for BMI, height and weight were calculated according to age and gender using the “growth analyser 3” developed by the Dutch growth foundation. We determined two SDS, one SDS_nonPWS_ using a healthy Dutch/German population as reference (2001 BMI for age Germany [26], 1998 height for age Germany [27], 1997 weight for age Netherlands [28]) and one disease specific SDS_PWS_ using a PWS population 2000 as reference [29]. Lean body mass (kg) and body fat content (%) were measured in all patients by body composition measurement from the age of 3 yrs. onwards and were given as absolute values. We used a Fresenius Medical Care Body Composition Monitor to study body composition. First, blood pressure, height, and weight were measured and then each subject was asked to lie down for a minimum of 5 min before the test in order to ensure optimum body water distribution. During the test, the monitor analyzed the body composition via bioimpedance spectroscopy. The relevant output parameters were obtained by means of validated physiological models. The electric conductance of a cell suspension, as described in the volume model, makes it possible to determine the volume of total body water as well as that of extracellular and intracellular water [30]. Therefore, overhydration, lean tissue, and adipose tissue mass can be calculated in a second step by using the information obtained beforehand about the extra- and intracellular water volume [31]. At the end of each test a quality between 0 (low quality) and 100 (optimum quality) was assigned. Only a test quality higher than 80 was accepted. Tests with lower quality were repeated under optimized test conditions.

#### Endocrine parameters

Quantitative serum IGF-I and IGFBP-3 values were determined with an immunometric assay (IMMULITE® 2000 systems, Siemens Healthcare Diagnostics Products Ltd., Caernarfon, United Kingdom). SDS was calculated manually according to age (IGF-I and IGFBP-3) and gender (IGF-I only) by employing reference intervals supplied by the manufacturer.

#### Lipid and carbohydrate metabolism

Triglycerides, high-density lipoprotein (HDL)-, low-density lipoprotein (LDL)-, total cholesterol, and insulin concentrations were measured by using commercially available kits from Siemens Healthcare Diagnostics. In order to measure triglycerides and total cholesterol levels enzymatically, the Dimension Vista® System Flex® reagent cartridge CHOL (AMR: 50–600 mg/dL [1.29–15.54 mmol/L]) and Dimension Vista® System Flex® reagent cartridge TRIG (AMR: 2–1000 mg/dL [0.02–11.30 mmol/L]) were employed. The Dimension Vista® System Flex® reagent cartridge LDLC (AMR: 1–300 mg/dL [0.03–7.77 mmol/L]) was used to determine LDL levels and the Dimension Vista® System Flex® reagent cartridge HDLC (AMR: 3–150 mg/dL [0.08–3.89 mmol/L]) was used to measure HDL directly by using a two reagent format. The IMMULITE® 2000 Insulin (Reportable Range: 2–300 μIU/mL) is a solid-phase, enzyme-labelled chemiluminescent immunometric assay and was employed to determine fasting insulin levels. HbA1c was determined immunochemically with high-performance liquid chromatography (LC Variant II Biorad®). Fasting glucose was measured with a UV-test (hexokinase-method with cobas 8000 c702 Roche®). Intra- and interassay coefficients of variation were below 5% with all methods. We employed the homeostasis model assessment of insulin resistance (HOMA-IR) index to estimate insulin resistance in our patients. HOMA-IR is defined as (G_0_ (mg/dl) * I_0_(μU/ml) / 405) whereby G_0_ is fasting glucose and I_0_ is fasting insulin [32]. All lipid and carbohydrate parameters were given as absolute values.

### Statistical methods

Differences between the early treatment Group A and the later treatment Group B in auxological, endocrine and metabolic parameters throughout the observation period were estimated with a linear mixed-effect model (MIXED), which defined patient as a random factor. In the regression framework, auxological, endocrine and metabolic parameters act as dependent variables determined by the independent variables age (continuous variable), group (categorical variable) and age by group interaction. In some cases, when the direction of the effect of the treatment group varied significantly depending on the observed age, an additional t-test for independent samples was performed for all ages. A t-test for independent samples was also employed to determine height-SDS_nonPWS_ development in group A and differences in GH dosages, BMI-SDS_PWS_ and baseline values between the groups. A *p*-value < 0.05 was considered significant. Statistical analyses were performed with SPSS Statistics 25 (SPSS Inc., Chicago, IL).

### Patients

This retrospective, longitudinal study included 62 children (31 males) with genetically confirmed PWS who started treatment with GH at the Endocrinology Department of the St. Bernward Hospital in Hildesheim, Germany between October 2007 and July 2015. Upon diagnosis, all children were offered GHT. Then, parents and physicians made a shared decision as to whether GHT should be started immediately or at a later time. In the course of GHT-monitoring, control examinations were performed regularly (August 2007 to August 2018). These included taking fasting morning blood samples and auxological parameters. Written informed consent was obtained from all parents and the study was approved by the ethics committee of the University Hospital of Bonn.

## Results

### Sample description

The early treatment cohort A consisted of 21 (11 males) infants with PWS who were recruited at the ages of 0.3–0.99 yrs. (mean 0.72 yrs) for initiation of GHT. The later treatment cohort B entailed 41 individuals (20 males) which started GHT at the ages of 1.02–2.54 yrs. (mean 1.42 yr). Fasting morning blood samples and auxological parameters were obtained at the ages of 0.5 (±0.25) yrs. (only Group A), 1 (±0.25) yr, 1,5 (±0.25) yrs., 2 (±0.25) yrs., 3 (±0.5) yrs., 4 (±0.5) yrs. and 5 (±0.5) yrs. In some patients, multiple observations were made in a short period of time. In those cases, only the sample taken closest in time to requested age was evaluated and the others were excluded from the study. At the age of 1 yr data were collected from 21 out of 21 children (100%) from Group A and from 32 out of 41 children (78%) from Group B. At the age of 5 yrs., fasting morning blood samples and auxological parameters were obtained from 11 children (52.4%) from Group A and from 39 children (95.1%) from Group B. Five patients dropped out for unknown reasons. The patients included in this study were primarily of German Caucasian descent and there were no significant numbers of patients with differing ethnic backgrounds represented in the sample. Although all participating children had genetically confirmed PWS (Table 1), the genetic subtypes were not determined in all cases. GH dosages did not vary significantly between the groups from 2 to 5 yrs. of age [5 yrs.: mean (± SD) A: 0.0289 (±0.005) mg/kg/d vs. B: 0.0263 (±0.006) mg/kg/d; *p* = 0.21]. From 1 to 1.5 yrs. of age significant differences between the two groups were determined [1 yr: A: 0.0203 (±0.012) mg/kg/d vs. B: 0.0008 (±0.004) mg/kg/d; *p* < 0,001; 1.5 yrs.: A: 0.0291 (±0.005) mg/kg/d vs. B: 0.0187 (±0.012) mg/kg/d; *p* < 0.001]. These differences are explained by slow titration of GH, especially in the later treatment Group B, until the mean dosages were reached for both groups. GHT was discontinued in one case because of the development of obesity hypoventilation syndrome.

**Table 1:** Genetic mutations of early treated (before 1st birthday; group A) and later treated (after 1st birthday; group B) patients

	Group A (*n* = 21)	Group B (*n* = 41)
Paternal deletion	*n* = 10 (47.6%)	*n* = 9 (21.95%)
Maternal uniparental disomy	*n* = 1 (4.8%)	*n* = 18 (43.9%)
Imprinting defect	*n* = 1 (4.8%)	*n* = 0 (0%)
Genetically confirmed PWS, no subtype	*n* = 9 (42.8%)	*n* = 14 (34.15%)

We performed a t-test comparing height-SDS_nonPWS_, BMI-SDS_nonPWS_ and LDL in group A at the age of 0.5 yrs. with group B at the age of 1 yr. The results showed no significant differences between the groups (height-SDS_nonPWS_: *p* = 0.310; BMI-SDS_nonPWS_: *p* = 0.908; LDL: *p* = 0.164).

### Anthropometry and body composition

**Fig. 1:**
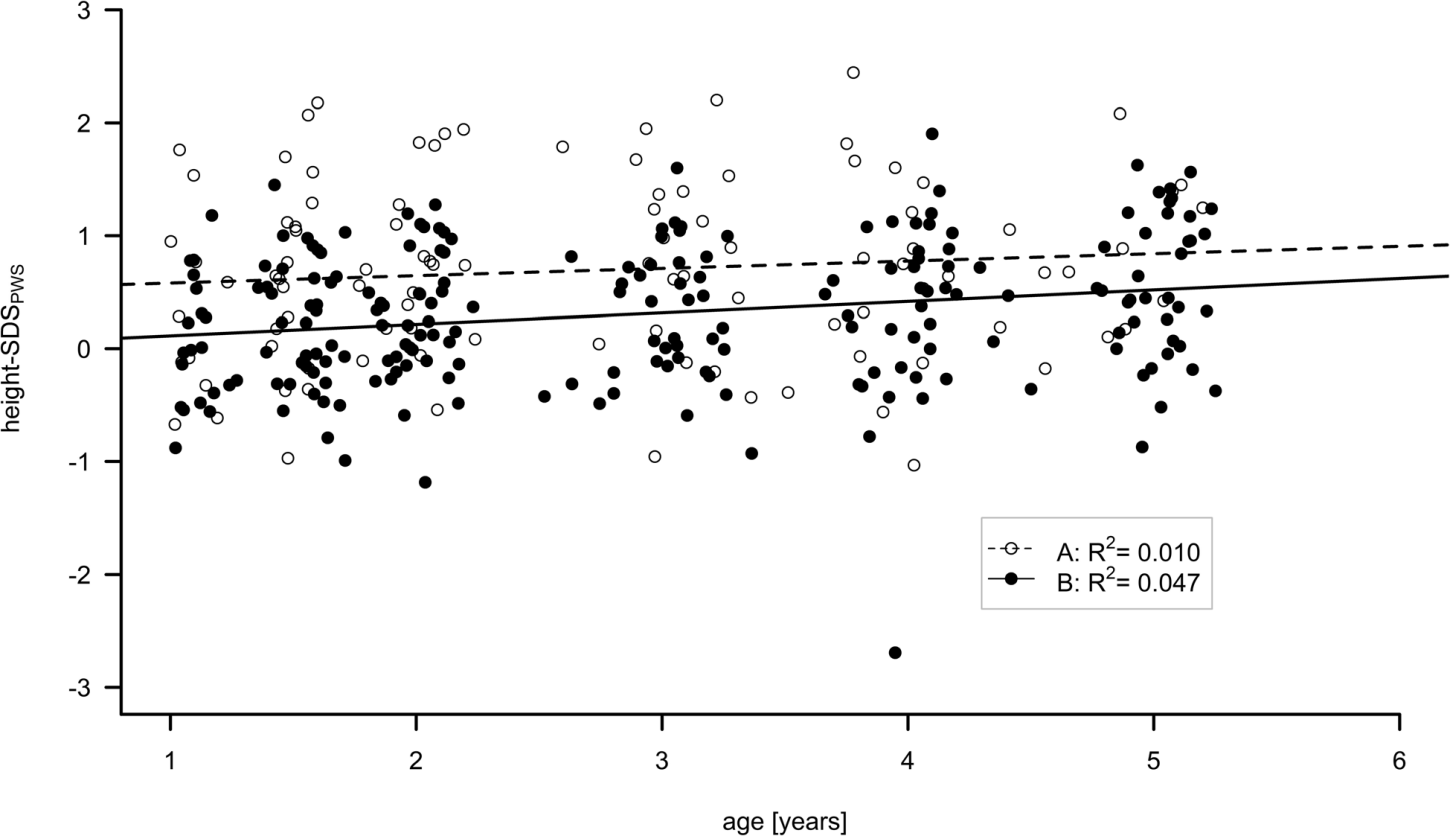
Length/height-SDS_PWS_ throughout the observation period from 0.5 to 5 year of age are shown. Open dots (individual data) and dashed line (regression line) represent PWS patients who were started on growth hormone therapy before the age of one. Filled dots and solid line those who were commenced on growth hormone after the first birthday

Mean length/height-SDS_PWS_ differed significantly throughout the entire observation period between the two groups (Fig. 1; mean length/height-SDS_PWS_ 1 yr: A: 0.37 (±0.83) vs B: 0.05 (±0.56); 5 yrs.: A: 0.81 (±0.67) vs. B: 0.54 (±0.64); *p* = 0.012). Mixed model analysis showed no evidence for an interaction between group and age (*p* = 0.344) with a parallel gain of 0.077 SDS per year of age (*p* < 0.001) and an advantage of 0.405 SDS for group A (*p* = 0.019). When height was measured at the age of 1 yr the early treatment Group A had already been treated from the mean age of 0.72 yrs. onwards. We performed a t-test comparing height-SDS_nonPWS_ in group A between the ages of 0.5 yrs. and 1 yr and found a significant difference (*p* = 0.040). The later treated Group B showed similar characteristics to an untreated PWS reference population in height at the age of 1 yr. Not only did the disease specific height SDS show that the earlier treated group was taller than the later treated group throughout the observation period, but the length/height-SDS_nonPWS_ values confirmed that as well (*p* = 0.049). We observed no significant differences in mean weight-SDS_PWS_ or in mean weight-SDS_nonPWS_ between the two groups throughout the study. Mean BMI-SDS_nonPWS_ increased throughout the observation period. This increase is, however, a natural part of the development of children with PWS and when looking at mean BMI-SDS_PWS_ during the course of the study a decrease can be observed in both groups. Whilst mean BMI-SDS_nonPWS_ did not show significant differences between the two groups (*p* = 0.360), mean BMI-SDS_PWS_ suggested slightly higher values in Group A from the age of 3 yrs. (*p* = 0.098). Yet, when tested individually for each age group, a t-test demonstrated no significant differences between the two groups in mean BMI-SDS_PWS_ from the age of 1.5 yrs. Solely at the age of 1 y, Group A showed a lower mean BMI-SDS_PWS_ than Group B (*p* = 0.044).

Our analysis presented no significant differences between the two groups in lean body mass (*p* = 0.261) or body fat content (*p* = 0.401). Both lean body mass and body fat values increased in both groups during the course of time.

### Endocrine parameters

Data are shown in Table 2. Mean IGFBP-3 SDS increased continuously (within the normal range) with 0.38 SDS per year in both groups but was 0.57 SDS higher (*p* = 0.001) in Group A compared to Group B throughout the observation period. This effect was steady and did not vary depending on age (*p* = 0.946). Mean IGF-I SDS in the early treatment Group A did not differ significantly from the later treatment Group B (*p* = 0.179) and mean IGF-I SDS were mostly below 0 SDS (within the normal range) in both groups during the course of the study.

**Table 2:** BMI, percentage of body fat, and endocrine data of early treated (before 1st birthday; group A) and later treated (after 1st birthday; group B) patients at all observational time points are shown. ^a^indicates a difference between the groups at the significance level of 0.05

Age	1 ± 0.25 yrs		2 ± 0.25 yrs		3 ± 0.5 yrs		4 ± 0.5 yrs		5 ± 0.5 yrs	
Group	A (*n* = 21)	B (*n* = 32)	A (*n* = 21)	B (*n* = 41)	A (*n* = 21)	B (*n* = 41)	A (*n* = 20)	B (*n* = 41)	A (*n* = 11)	B (*n* = 39)
mean ± SD										
BMI_nonPWS_-SDS	−1.73 ± 1.41	−0.99 ± 1.32	−1.04 ± 1.20	−0.88 ± 1.20	−0.16 ± 1.10	−0.62 ± 1.49	0.15 ± 1.16	0.02 ± 1.36	0.22 ± 0.92	0.18 ± 1.18
BMI_PWS_-SDS	−0.49 ± 0.74	0.11 ± 0.77	−0.68 ± 0.69	−0.61 ± 0.71	−0.85 ± 0.77	−1.10 ± 0.86	−1.01 ± 0.81	−1.12 ± 0.95	−1.26 ± 0.64	−1.30 ± 0.82
% body fat					14.74 ± 6.24	15.06 ± 5.54	17.52 ± 8.59	19.41 ± 6.57	19.42 ± 5.32	21.10 ± 4.80
IGF-I SDS	−0.62 ± 1.62	−0.57 ± 1.69	−0.30 ± 2.02	0.32 ± 2.53	−0.86 ± 2.21	−0.57 ± 2.07	−0.74 ± 1.78	−0.47 ± 1.97	−0.35 ± 2.45	−0.98 ± 1.25
IGFBP-3 SDS	0.25 ± 0.71^a^	−0.79 ± 0.69^a^	1.22 ± 0.76^a^	0.58 ± 0.96^a^	1.31 ± 0.78^a^	0.89 ± 0.91^a^	1.46 ± 1.03^a^	0.99 ± 0.81^a^	1.12 ± 1.19^a^	0.97 ± 0.91^a^

### Lipid metabolism

**Fig. 2:**
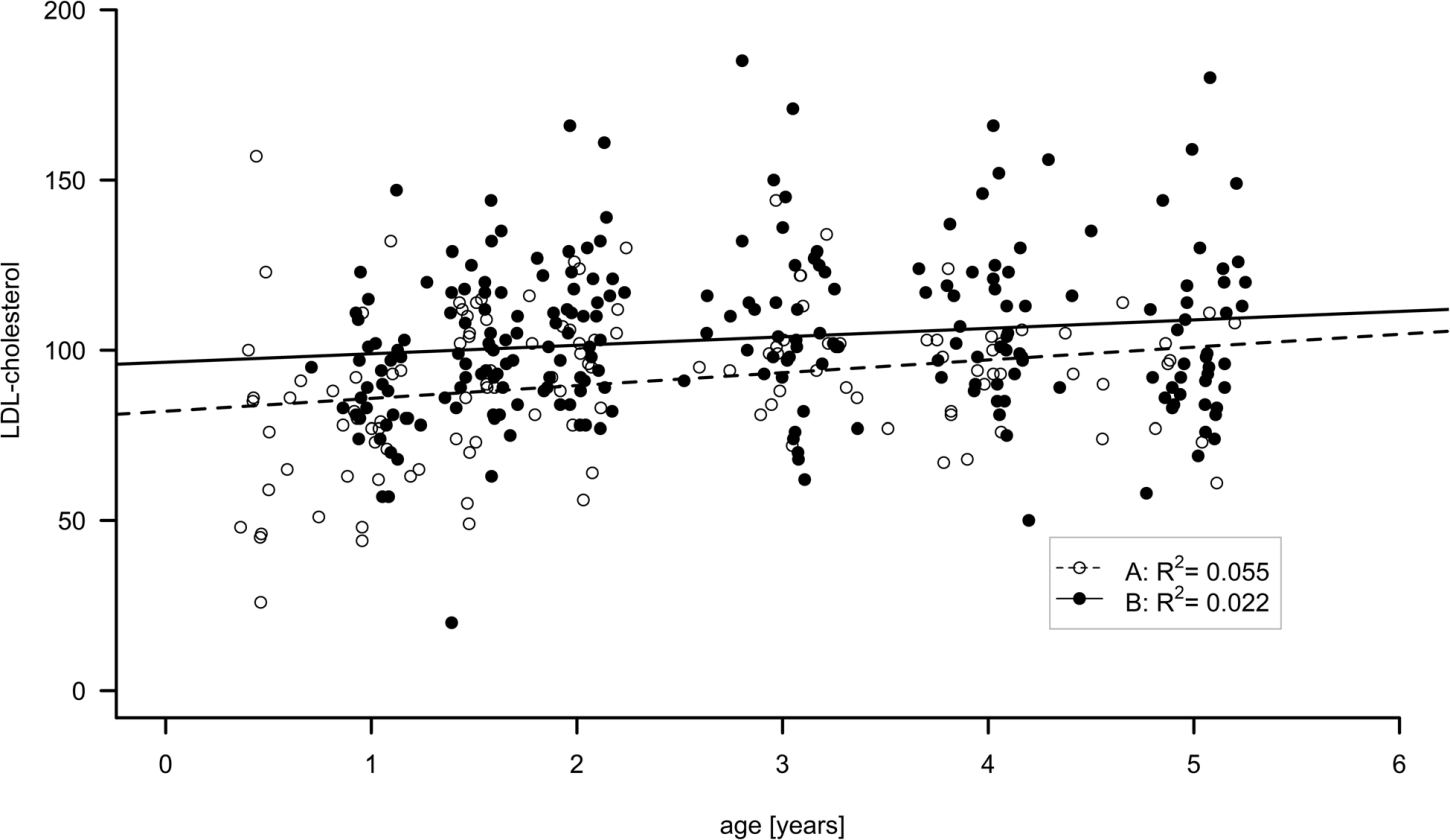
Individual LDL cholesterol concentrations throughout the observation period from 0.5 to 5 year of age are shown. Open dots (individual data) and dashed line (regression line) represent PWS patients who were started on growth hormone therapy before the age of one. Filled dots and solid line represent those who were commenced on growth hormone after the first birthday

Low-density cholesterol (LDL) was statistically significantly lower in Group A than in Group B during the entire course of the study (Fig. 2; LDL: 1 yr: A: 79 (±20) mg/dl vs. B: 90 (±19) mg/dl; 5 yrs.: A: 91(±18) mg/dl vs. 104 (±26) mg/dl; *p* = 0.024). Furthermore, we observed a trend towards lower total cholesterol (TC) values in Group A as against those in Group B throughout the observation period [TC: 1 yr: A: 136 (±17) mg/dl vs. B: 148 (±22) mg/dl; 5 yrs. A: 159 (±30) mg/dl vs. B: 174 (±29) mg/dl; *p* = 0.077]. Our analysis showed that the effect of the treatment group on LDL levels was steady and did not vary significantly in the course of the observation period (*p* = 0.600). We observed no significant differences in high-density cholesterol (HDL) levels between the two groups (*p* = 0.377). Triglycerides were not significantly different between the early treatment Group A and the later treatment Group B [1 yr: A: 74 (±26) mg/dl vs. B: 84 (±35) mg/dl; 5 yrs.: A: 59 (±15) mg/dl vs. B: 72 (±25) mg/dl; *p* = 0.548].

### Carbohydrate metabolism

**Fig. 3:**
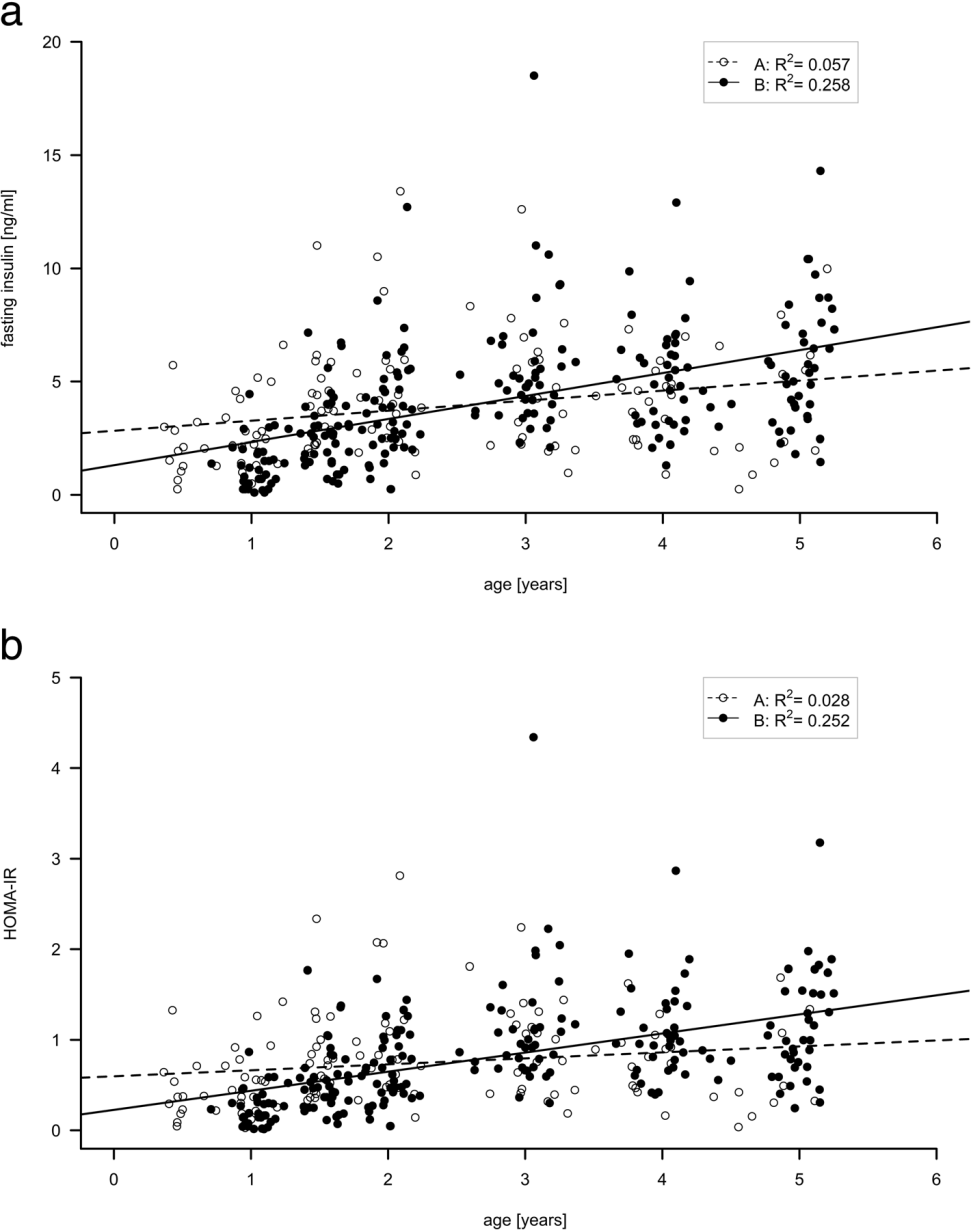
**a** Individual Insulin concentrations throughout the observation period from 0.5 to 5 year of age are shown. Open dots (individual data) and dashed line (regression line) represent PWS patients who were started on growth hormone therapy before the age of one. Filled dots and solid line those who were commenced on growth hormone after the first birthday. **b** HOMA Index throughout the observation period from 0.5 to 5 year of age is shown. Open dots (individual data) and dashed line (regression line) represent PWS patients who were started on growth hormone therapy before the age of one. Filled dots and solid line those who were commenced on growth hormone after the first birthday

Data are shown in Fig. 3a (fasting insulin) and 3b (HOMA-IR). Differences in the development of mean fasting insulin levels and HOMA-IR between the two groups were found (group by age interaction fasting insulin *p* = 0.001; HOMA-IR p = 0.001). We performed an additional t-test to analyze the differences between the two groups for all ages. At the age of 1 to 1.5 yrs. statistically significantly higher mean fasting insulin levels and HOMA-IR were measured in Group A [1 yr: fasting insulin: A: 2.53 (±1.75) ng/ml vs B: 1.27 (±1.04) ng/ml (*p* = 0.006); HOMA-IR: 1 yr: A: 0.51 (±0.38) vs. B: 0.24 (±0.20); *p* = 0.005]. During the course of the study, the effect reversed leading to significantly higher HOMA-IR levels in Group B from the age of 4 yrs. onwards and to significantly higher fasting insulin levels at the age of 5 yrs. [5 yrs.: fasting insulin A: 3.99 (±3.18) ng/ml vs. B: 5.75 (±2.75) ng/ml; *p* = 0.076; 5 yrs.: HOMA-IR A: 0.65 (±0.58) vs. B: 1.13 (±0.59); *p* = 0.034]. Significant differences in HbA1c and blood glucose levels were also determined between the two groups (HbA1c: *p* < 0.001; blood glucose: *p* = 0.022). The treatment effect varied significantly depending on the observed age (HbA1c: *p* < 0.001; blood glucose: *p* = 0.094) which again led us to perform a t-test. In line with our results displaying the development of fasting insulin levels and HOMA-IR, the t-test showed that significantly higher mean HbA1c and blood glucose values were measured from 1 to 1.5 yrs. of age in Group A [1 yr: HbA1c: A: 4.81 (±0.70) % vs. B: 3.98 (±0.85) %; *p* = 0.001; 1 yr: blood glucose: A: 78.71 (±12.92) mg/dl vs. B: 72.50 (±12.30) mg/dl; *p* = 0.084]. During the course of the study, HbA1c and blood glucose values measured in both groups converged and no more significant differences were determined from the age of 1.5 yrs. and older.

## Discussion

This longitudinal, retrospective study of GHT in 62 children with PWS compared one group with very early GHT onset during their first year of life to a second group of patients which commenced GH-therapy after their first birthday. Remaining within the normal reference ranges throughout the observation period, our analysis showed that early onset of GHT had a statistically significant favorable effect on height-SDS, LDL cholesterol, HOMA-IR and fasting insulin. The two groups did not differ in BMI-SDS, body composition or IGF-I SDS.

Children with PWS have impaired growth and, consequently, only attain an adult height below the 3rd centile [29]. In accordance with the literature [15, 17, 21, 33] we found a complete normalization of mean height-SDS in both groups when compared to a non-PWS reference population. Mean height-SDS in the earlier treatment Group A was, however, significantly closer to 0 SDS_nonPWS_ than in Group B throughout the observation period. As mentioned in van Lind Wijngaarden et al. mean height-SDS, although normalized, did not rise above 0 SDS_nonPWS_ [15, 17]. Nevertheless, higher values were seen in individual patients in both groups. Thus, our results indicate that very early treatment onset with GH results in mean height-SDS more similar to those of a non-PWS reference population than onset after the first year of life. This finding is in line with most publications [16, 17, 19, 21, 34]. Seeing as our patient group was only observed until the maximum age of 5 yrs., it has yet to be shown whether this effect remains until they reach their final height. BMI-SDS_PWS_ decreased in both groups throughout the observation period but no significant differences were determined between the early treatment Group A and Group B. Mean BMI-SDS_nonPWS_ remained within the lower part of the normal range (< 0,5 SDS) throughout the years in both groups but increased continuously within the lower part of the normal range with time. This trend could also be observed in the increase of body fat content in both groups during the course of the study. Our analysis showed no differences between the groups in body fat or lean body mass. According to Festen et al. [16] GHT prevents the loss of lean body mass normally seen in non-GH treated children with PWS during the course of time, even if lean body mass adjusted to height does not normalize or increase. Low lean body mass in PWS probably represents reduced muscle mass and may therefore be linked to clinical hypotonia, reduced physical performance and, consequently, reduced energy expenditure [35].

Children with PWS are highly sensitive to GHT [36]. Given that high IGF-I levels have potentially been linked to adverse events [37, 38], biomonitoring is recommended in order to keep IGF-I levels within the upper part of the normal range (+ 1 to + 2 SDS) [14]. In our study, mean IGF-I SDS were in the normal range, mostly below 0 SDS in both groups. This may be due to the use of relatively low GH dosages. The dosage of 1 mg/m^2^/d (~ 0.035 mg/kg/d) is recommended by the consensus guidelines for rhGH therapy in PWS [14]. In a cross-sectional study, Bakker et al. found no correlation between serum IGF-I levels and IGF bioactivity in GH treated PWS-children and therefore suggested that IGF-I levels are an inappropriate method for GH dosing [39]. Instead, a recent study proposed the use of bioactive IGF-I as a more effective monitoring parameter [40]. This should be kept in mind for future studies. According to the dual effector hypothesis [41], growth promoting effects of GH have been explained by direct effects of GH on peripheral tissues not mediated by IGF-I on the one hand, and by GH-stimulated IGF-I production for autocrine/paracrine (A/P) action as well as endocrine acting circulating IGF-I (mostly due to stimulation of IGF-I production by the liver and other tissues [42]) on the other hand. Festen et al. found that neither height-SDS nor BMI-SDS were correlated to IGF-I SDS [16]. Feigerlová et al., however, determined significant relationships between these parameters up until the completion of 12 months of treatment but not at 24 months after GHT onset [36]. In our study, height-SDS differed significantly between the groups whereas IGF-I SDS did not.

IGFBP-3 SDS values were within the normal range in both groups, but significantly higher (within the upper part of the normal range) in the earlier treatment Group A. This is in accordance with other studies [15, 16, 36] describing rising IGFBP-3 levels within the normal range in response to GHT.

Our analysis showed that patients treated with GH before their first birthday had more favorable mean LDL cholesterol levels than later treated children with PWS. Positive effects of GHT on LDL in children with PWS in general have been reported by various authors [17, 19–21]. In contrast, van Lind Wijngaarden [34] found no effect of GHT on LDL but described an improved HDL-LDL ratio in agreement with findings by l’Allemand et al. [20]. There are conflicting results as to whether HDL- cholesterol levels are improved [19, 20] or not [15, 17, 21, 34] by GHT. In our study, there was no significant difference in HDL values between the two groups. However, our analysis demonstrated a trend that an early treatment onset decreases the total cholesterol values. A positive effect of GHT on total cholesterol was described by various authors [15, 19, 21], in contrast, no effect on total cholesterol was observed by van Lind Wijngaarden et al. [17]. Triglyceride levels did not differ significantly between both groups in our study. This is in line with other publications reporting unchanged triglyceride levels during GHT [15, 19]. In non-syndromal obesity, fat mass and especially visceral fat mass determine serum lipid levels [43]. Yet, L’Allemand et al. [20] found no correlation between total body fat or trunk fat mass with LDL in children with PWS and suggested that changes in cholesterol levels during treatment depend on the effects of GH on the lipid metabolism itself. This is in agreement with observations made in a study with GH-deficient adults in which favorable effects of GHT on cholesterol levels were not associated with adiposity [44]. Rudling et al. found that GH has a direct effect on the expression of hepatic LDL-receptors regulating the amount of circulating LDL-C [45]. Liu et al. also reported direct effects of GH on lipid uptake and de novo lipogenesis [46]. In accordance with the publications mentioned above, our analysis showed no differences in BMI-SDS or body fat content between the two groups but a significant difference in LDL levels. In line with publications describing an altered lipid metabolism during the course of GHT in children with PWS, our analysis shows that even earlier treatment onset can further influence LDL and, possibly, total cholesterol levels. Considering cardiovascular diseases as one of the main causes of death in adults with PWS [47], these findings are highly relevant for patients.

Children with PWS are more insulin sensitive and less insulin resistant than children with non-syndromal obesity [48]. This may be due to the fact that PWS patients have relatively low visceral adiposity compared to simple obese patients [49]. Still, adults with PWS exhibit a high incidence of T2DM [50]. Thus, the question arises, whether the diabetogenic effect of GHT further impairs the glucose homeostasis in patients already prone to develop diabetes. Various authors have reported no effect of GHT in children with PWS on the carbohydrate metabolism [17, 19, 34]; one possible explanation is that the long-term beneficial effects of GHT on body composition outweigh the GH-induced reduction of insulin sensitivity [14, 21]. Yet, our analysis showed that HOMA-IR and fasting insulin values were significantly higher in the early treatment Group A than in Group B until the age of 1.5 yrs. Bakker et al. [21] also reported rising fasting insulin levels and HOMA-IR during the first year of GHT. Levels then remained stable in the following years and at 8 yrs. were not significantly different from those 1 yr after GHT onset. Other publications describe a similar effect during the first year of GHT [15, 51]. In our study, Group A was treated earlier and, in accordance with the literature, fasting insulin and HOMA-IR rose to higher levels in Group A’s first treatment year than in the mostly untreated Group B. Once GHT was started in Group B values no longer differed between the groups. Yet, from the age of 4 yrs. onwards HOMA-IR was significantly lower in Group A than in Group B and at the age of 5 yrs. fasting insulin levels were significantly lower in Group A. Several authors [17, 21] found that body fat content is associated with fasting insulin and HOMA-IR, underlining the importance of maintaining a normal BMI in children with PWS. Our analysis, however, showed no differences between Group A and B in body fat or BMI-SDS, suggesting further GH-induced mechanisms are involved in the glucose metabolism. Our findings are in line with Yuen et al., who showed that low doses of GH treatment enhanced insulin sensitivity in GH deficient adults and improved peripheral glucose uptake [52]. Boparai et al. reported beneficial effects on the glucose metabolism in GH-transgenic mice with increased systemic GH levels and suggested a compensation through an increase in insulin release as the cause [53]. Our study does not only affirm the lack of GH-induced adverse effects on glucose metabolism but shows substantial beneficial effects of GHT on HOMA-IR and fasting insulin levels. Thus, our results indicate that children with PWS might benefit from early GHT initiation during their first year of life in terms of the glucose metabolism.

Further research is necessary to investigate the exact effects GH has on the carbohydrate metabolism in children with PWS. Our analysis was concluded when the children reached the age of 5 yrs. Therefore, it is imperative that future studies determine whether the observed effects persist into puberty and adulthood and whether the cessation at this point leads to a deterioration of metabolic parameters and body composition. Koizumi et al. reported an increase in LDL and visceral body fat 6 months after cessation of GHT in adults with PWS [54]. Kuppens et al. [55] also described increases in fat mass when GHT was discontinued in young adults and therefore suggested there were potential benefits in continuing GHT even after adult height is reached.

One major strength of this study was its longitudinal evaluation between 1 and 5 yrs. of age. Another strength was the large study cohort compared to those of previous publications.

To our knowledge, this was the first study evaluating whether an age above or below 1 year at GHT start influences auxological, endocrine and metabolic parameters. Mean treatment onset in Group B was 0.7 yrs. later than in Group A, thus, GHT was also commenced relatively early in our later treatment cohort.

One limitation was the study’s retrospective design and an incongruent number of patients per group, in particular a drop in participants from the fourth to the fifth year analysis timepoint; which should be adjusted in future studies. The patients participating in this study were primarily of German Caucasian descent; accordingly, future studies should investigate the effects in different cohorts. Although the literature reports that PWS genetic subtypes are mainly linked to characteristic physical appearances and behavioral differences [56, 57], future studies should analyze possible metabolic differences between the genetic subtypes and their response to GHT.

Lean body mass and body fat were not measured with DEXA and may, therefore, be imprecise. In future studies, these parameters could also be adjusted to height and sex. Further possible improvements may also be include the use of PWS reference values in more parameters than height, weight and BMI and the comparison with a non-PWS control group.

## Conclusion

This study compared auxological, endocrine and metabolic parameters in children with PWS treated with GH before and after their first birthday. Both groups were treated from an early age onwards and were given relatively low GH dosages. Our results show that early GH onset during the first year of life has statistically significant favorable effects on height-SDS, lipid profile and glucose metabolism. Therefore, earlier GHT onset should be recommended to families with children with PWS.

## Data Availability

The datasets used and/or analyzed during the current study are available from the corresponding author on reasonable request.
